# Improving the mental wellbeing of Arabic speaking refugees: an evaluation of a mental health promotion program

**DOI:** 10.1186/s12888-020-02732-8

**Published:** 2020-06-18

**Authors:** Shameran Slewa-Younan, Molly McKenzie, Russell Thomson, Mitchell Smith, Yaser Mohammad, Jonathan Mond

**Affiliations:** 1 Mental Health, Translational Health Research Institute, School of Medicine, Humanitarian and Development Research Initiative, Western Sydney University, Campbelltown, Australia; 2 Centre for Mental Health, Melbourne School of Population and Global Health, University of Melbourne, Melbourne, Australia; 3 Mental Health, School of Medicine, Western Sydney University, Campbelltown, Australia; 4 School of Computing, Engineering and Mathematics, Western Sydney University, Sydney, Australia; 5 NSW Refugee Health Service, South Western Sydney Local Health District, Liverpool, NSW Australia; 6 Centre for Rural Health, College of Health and Medicine, University of Tasmania, Hobart, Australia

**Keywords:** Refugee, Mental health, Mental health literacy, Mental health promotion

## Abstract

**Background:**

Refugee populations have particularly high rates of mental health problems, including Posttraumatic Stress Disorder (PTSD) and depression. However, uptake of mental health care may be low even when severe depression and PTSD symptoms are present in individuals following resettlement. This is likely due, at least in part, to cultural influences on refugees’ knowledge and beliefs about mental health problems and their treatment. We sought to provide preliminary evidence for the effectiveness of a culturally tailored mental health promotion program for Arabic-speaking refugees.

**Methods:**

A total of 33 Arabic-speaking refugees resettled in South Western Sydney were recruited and completed intervention which consisted of weekly three-hour sessions for 4 weeks delivered in Arabic. Key aspects of mental health literacy, help-seeking intentions and levels of general psychological distress were assessed, by means of a self-report survey, pre-intervention, (immediately) post-intervention and 3 months following intervention.

**Results:**

Of the 33 participants that completed the intervention, 31 completed the immediate post-intervention survey and 29 completed the 3 months follow-up survey. Improvements in most aspects of mental health literacy assessed were found immediately post-intervention and at follow-up, although only changes relating to stigmatising attitudes were statistically significant. Additionally, a statistically significant decrease in participants’ levels of general psychological distress was observed immediately following the intervention, and this decrease was sustained at follow-up.

**Conclusion:**

While further research employing a more rigorous study design and larger sample size will be needed, results of this initial trial suggest that a culturally tailored mental health promotion program targeting key aspects of mental health literacy can improve the mental health of Arabic-speaking refugees resettled in a Western nation.

## Background

The number of people being forcibly displaced from their homes is increasing, with the United Nations High Commission for Refugees (UNHCR) figures indicating 16.2 million newly displaced people in 2017 alone [1]. In the period of 2015–2016 a total of 17,555 refugees were resettled in Australia [2]. According to the Australian Bureau of Statistics (ABS) 2016 census, Iraq is the country of origin of the highest number of permanent humanitarian migrants, accounting for 17.6% of all such migrants in Australia [3].

High prevalences of Post-Traumatic Stress Disorder (PTSD) and depression in resettled refugee populations are well-established [4, 5]. A 2005 review of 20 studies of refugees resettled in Western countries showed a 10 fold increase in PTSD in these populations when compared with general population data [6]. A 2014 review of Iraqi refugees resettled in Western countries found rates of PTSD between 8 and 37.2% and rates of depression between 28.3 and 75% [5], levels of these conditions far exceeding those observed in general population samples in both the USA and Iraq [5].

Post-migration, refugees must deal with many issues, including cultural dislocation, racism, financial and housing insecurity, separation from family and limited social support [5, 7]. These challenges compound adverse effects of pre-migratory trauma on mental health, and evidence, including a recent study of Iraqi refugees resettled in Australia, suggests that a longer period since resettlement can lead to higher levels of psychological distress [7]. As such, pre-migratory traumatic events combined with post-migration and resettlement stressors put refugee populations at high risk for the development of trauma-related mental health problems [6].

Research has demonstrated that “mental health literacy” (MHL), including knowledge and beliefs about the nature and management of mental health problems, help-seeking and support services available [8–12], may be particularly problematic in resettled refugee populations, and that this is a barrier to help being received when it is needed. In a study of Iraqi refugees resettled in Australia, employing a vignette methodology, only 14.2% of Iraqi participants correctly identified PTSD as the problem depicted in the vignette, compared to 34.3% in the 2011 National Survey of Mental Health Literacy and Stigma (NSMHLS) [8]. Further, only 19% of participants in this research reported seeking help for a mental health problem, despite high levels of PTSD symptoms and general psychological distress [10]. Similar findings have been reported in previous studies of refugees resettled in Australia where hospital admissions data in Victoria demonstrated that refugee populations were much less likely to have hospital admissions for mental/behavioural disorders than the Australian-born population sample [13].

In spite of these findings, few trials of programs designed to improve MHL in resettled refugee populations have been conducted. As we, and others, have noted [14], there are significant challenges involved in developing and implementing programs of this kind, for example, differing views of interpreting and expressing psychological distress which can influence help-seeking behaviours and sources of help sought. To the best of our knowledge, we were able to identify three programs that have direct relevance to this study, as they sought to either improve MHL or address aspects of help-seeking in refugee populations [15–17]. Sanhori et al. [15] undertook a longitudinal study assessing 1529 internally displaced persons from two randomly selected areas in central Sudan. Participants were provided a short (four-hour) psychoeducational intervention, in which information about mental health, symptoms, treatment and mental-health-related stigma was presented [15]. Using a six item scale examining stigma and social distance levels, a follow up interview was completed 1 year after the baseline measure, with no significant reductions in stigma or social distance being noted [15]. A second recent study involved pre-post assessment of the effects of a six-day spirituality education program among diverse groups of refugees (*n* = 4504) in 38 camps within European nations [16]. The program focused on identifying mental health disturbance, addressing emotions, developing skills in centring, invoking a sense of calmness, and provision of instruction on mindfulness and wellbeing [16]. Pre and post intervention measures of trauma symptomology, levels of optimism and general psychological wellbeing were made. Authors noted an overall improvement across all three measures and pointed out that the most significant improvement was found in participants who self-reported willingness to practice the mindfulness and centring exercises [16]. Finally, Subedi et al. [17] reported on the impact of a 1 day mental health first aid (MHFA) training program delivered to Bhutanese refugee participants based in the United States. A total of 58 participants completed a pre and post- training survey which was a culturally adapted version of the MHL instrument developed for MHFA training in Australia [17]. Surveys were completed immediately prior to and after the MHFA intervention. The assessment included a vignette describing a person suffering depression followed by questions assessing knowledge and attitudes about mental health conditions and questions regarding post-resettlement stressors. Significant improvement was shown in correct identification of mental health conditions, knowledge of treatment options for the mental health problem in the vignette, and confidence relating to the provision of support for individuals suffering from mental health problems. However no change was observed for stigmatising attitudes [17].

Findings from the abovementioned studies suggest that positive results from mental health promotion programs designed to improve MHL in resettled refugee populations are possible, and with improved MHL, greater help-seeking intentions and behaviours may enable mental health needs to be addressed at an early and appropriate level. However, there is a need for further research in this field. In particular, efforts to develop an intervention program tailored to address the influence of unique cultural, religious and social values on the mental health literacy and help-seeking of specific groups of refugees remains lacking, and was a motivating factor for this study. Thus, the goal of the current study was to expand the current evidence base by conducting a preliminary trial of a culturally tailored mental health promotion program designed to improve MHL among two Arabic-speaking refugee populations in South Western Sydney, Australia.

## Methods

### Participants

**Fig. 1:**
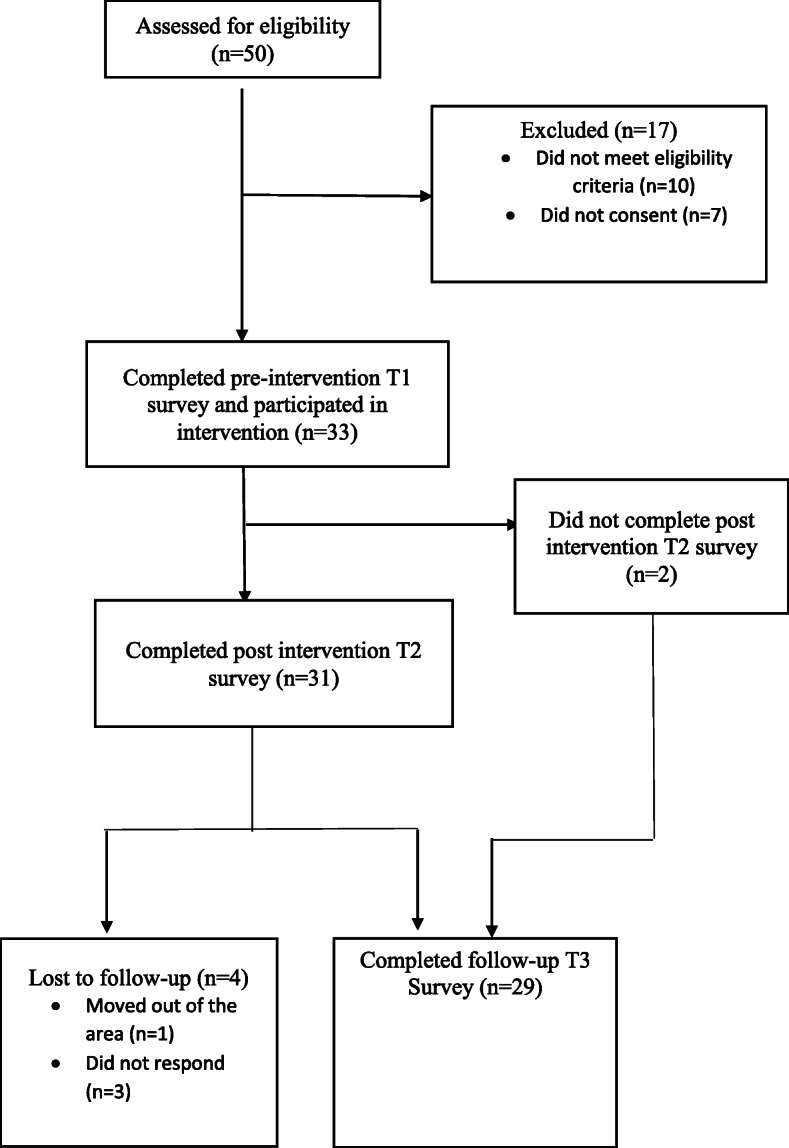
Participant flow throughout research stages

A total of 33 participants were recruited from an Adult Migrant English Program (AMEP) in South Western Sydney. Eligibility criteria included Arabic-speaking women and men, aged 18 years or more, born in Iraq or Syria and who had arrived in Australia as under the Humanitarian Migration Program. Data for the 31 participants who completed at least two of the three assessments were analysed (see Fig. 1). All study materials - participant information sheet, consent forms, program materials, slides and survey forms – were provided in Arabic. Participants were informed that participation in the program might lead to feelings of distress, that participation in the program was on a voluntary basis and that as such they were free to discontinue participation at any time. Participants who completed the program and assessments received an $80 supermarket voucher as reimbursement for their time and efforts. Ethics approval was granted by the Western Sydney University Human Research Ethics Committee (approval no. H12707).

### Intervention

The program entailed attendance at weekly sessions, each of 3 hours duration, over a four-week period delivered in Arabic by experienced bilingual health educators and/or mental health clinicians. The program content, which was developed by the authors in partnership with the NSW Refugee Health Service, was designed to be culturally sensitive and to be interactive with group discussion encouraged. The program was informed from the findings of our previous work on the MHL of refugees [8, 11, 12] where it was noted that a duality or pluralism of treatment beliefs amongst refugee groups exists and should be incorporated to enhance engagement. The concept of pluralism in psychological theory is not new, initially discussed by William James in 1908 but more recently offered as a stance in counselling and psychotherapy, which states that the client’s knowledge needs to be taken seriously [18]. In working with refugee populations, where health and treatment beliefs can be influence by religion and culture, the need to identified their preferred beliefs and practices is essential to engagement. More specifically, the importance of religion in shaping and influencing the beliefs towards mental illness and help-seeking in Arab populations and other closely aligned communities cannot be underestimated [19]. As such it was deemed necessary that the mental health literacy and promotion program should embrace the positive messages from religious teachings while targeting the areas for improved knowledge. For example, when developing sessions on knowledge around mental health treatment approaches used in Australia and its mental health systems, this was interwoven with culturally sensitive practices such as seeking spiritual advice, guidance and prayer, preferences previously highlighted in our MHL of refugees investigations [8, 11, 12]. Session two, which focussed on common mental disorders in refugee populations, also discussed differing perceptions of psychological distress amongst cultural groups. With respect to process, the cultural sensitivity and relevance of the developed sessions was undertaken through a working group comprising of experts on Iraqi and Syrian mental health (*n* = 2), mental health and health promotion (*n* = 1) and refugee health more broadly (*n* = 1). Additionally, two community elders from the Iraqi and Syrian community (*n* = 2) provided guidance and feedback on the final developed sessions. Broadly, four key components addressed in the respective sessions, were: exploring ‘good mental health’ and ‘wellbeing’; education about common mental health disorders (Depression, PTSD, Anxiety and Panic Attacks) and their presentation in refugee populations; explanation of the mental health system in Australia; and self-help strategies to promote good mental health including an introduction to mindfulness and relaxation. Table 1 outlines details of the structure and content of the program.

**Table 1:** Structure and content of the mental health promotion program for Arabic-speaking refugees

Session One: Introduction and covering the core concept of what is good mental health and wellbeing	Session Two: Mental health problems and illness	Session Three: Stigma and help-seeking	Session Four: Self-help strategies
Introduction of facilitator and goals of the program	Recap previous session	Recap previous session	Recap previous session
Ice-Breaker activity	Define mental illness and flag common mental disorders in refugee populations	Early intervention and help-seeking barriers	Everyday actions to promote wellbeing
Discuss ‘Good Mental Health’ and Mental Wellbeing	Group activity	Group activity	Discuss improving sleep hygiene
Breakout group activity	Common mental disorders in refugee populations	Reducing stigma and the Australian mental health care system	Mindfulness exercise
Summary of good mental health and wellbeing	Summary of mental illness	Summary of help seeking and the importance of early intervention	Summary of the content of all previous weeks and completion of post intervention survey

### Survey measure

A self- report survey that included items assessing key aspects of MHL as outlined below, help-seeking intentions and general psychological distress, was developed based on previous work by the authors and by Jorm and colleagues [8, 20]. The survey was administered pre-intervention, immediately post-intervention (i.e., upon completion of session four), and as a 3-month follow-up. A measure of psychological distress was included in order to permit assessment of whether any observed improvements in MHL were accompanied by improvements in mental health per se. Questions addressing demographic characteristics, namely, age, gender, marital status were also included.

### Assessment of mental health literacy

At the start of the survey, participants were provided with a vignette depicting a fictional character, named Dawood or Miriam (the gender of the character being matched to that of the participant), who was a refugee from Iraq suffering symptoms of PTSD according to the DSM-5 [21]. A series of questions, as outlined below, followed. The vignette was developed by the authors and has been used in several previous studies [8, 10–12, 22, 23].

Mental health problem recognition was assessed first. Participants were asked ‘What would you say is Dawood/Miriam’s main problem?’ and were required to select one option from a list of 11 possible options. Responses of ‘Fear’, ‘Stress-Related Disorder’ or ‘PTSD’ were coded as “correct” responses, while other responses (e.g., homesick, brain tumour, not a real problem) were coded as “incorrect”. Participants were next asked to rate the perceived helpfulness (‘helpful’, ‘harmful’, ‘neither’) of different possible interventions - actions/activities, medications and treatment providers - for someone with Dawood’s /Miriam’s problem, as well as which option in each category would be ‘most helpful’. Interventions were classified as being concordant with evidenced-based treatment of PTSD using the framework developed by Morgan and colleagues [24]. The extent to which interventions were culturally and spiritually informed was also considered, given past research has indicated the importance of dual treatment preferences in refugee populations [8, 9, 11]. Examples of interventions deemed to be consistent with evidence-based treatment were cognitive behaviour therapy, seeing a general practitioner, psychologist or psychiatrist, and taking anti-depressant medication, while examples of interventions deemed to be culturally and spiritually informed were reading the Koran or Bible, attending a prayer session or reading with a religious leader, and attending an Iraqi/Syrian social group or club.

### Negative attitudes towards mental illness

Participants’ negative attitudes towards mental illness were assessed using the modified Personal Stigma in Response to Mental Illness Scale [22, 25, 26]. Personal stigma was assessed by asking participants to respond to nine statements concerning the person described in the vignette using a 5-point Likert-type scale (1: ‘strongly agree’ to 5: ‘strongly disagree’). For purposes of analysis, the statements were divided into three components; ‘weak-not-sick’, ‘I would not tell anyone’ and ‘dangerous/unpredictable’ subscales, as previously used and validated [27]. The ‘weak-not-sick’ subscale focuses on the belief that the person is not ill and can control their behaviour (e.g. ‘Dawood/Miriam could snap out of it if he/she wanted to’). The ‘I would not tell anyone’ subscale focuses on the belief it is better not to tell anyone about mental illness (e.g. ‘You would not tell anyone if you had a problem like Dawood/ Miriam’s). The ‘dangerous/unpredictable’ subscale focuses on the belief that someone with a mental illness is dangerous or unpredictable (e.g., ‘Dawood/Miriam’s problem make him/her unpredictable’). Lower scores indicated greater personal stigma for that component.

Social distance was assessed using five statements from the social distance scale developed by Link and colleagues [28], which has also been used in previous research by the authors [22]. Participants were asked to consider whether/to what extent they would be pleased to spend time with Dawood/Miriam in different situations: ‘living next door to Dawood/Miriam’ and ‘having Dawood/Miriam marry into your family’, for example. Responses to these items were scored on a 4-point Likert scale ranging from 1 (‘Yes, definitely’) to 4 (‘Definitely not’). A total social distance score was calculated as the sum of responses to the individual items, with higher scores indicating greater desire for social distance.

### Help-seeking intentions

Help-seeking intentions were assessed by asking participants who they would be most likely to approach first for help if they had a problem such as the one described in the vignette. Participants were asked to select one of 13 options, examples being a general practitioner, psychologist, family member, religious leader or priest. Responses to this question were also classified as being consistent or not consistent with evidence-based treatment of PTSD [24] and culturally and spiritually informed practices.

### General psychological distress

Participants’ levels of general psychological distress were assessed using the 10-item scale developed by Kessler and colleagues [29] (K-10). Items of the K-10 assess the frequency of occurrence of each of 10 common symptoms of anxiety and depression during the past 4 weeks. Scores on these items range from 1 (‘none of the time’) to 5 (‘all of the time’). Total scores therefore range from 10 to 50, with higher scores indicating higher levels of distress. Total scores in the ranges of 10–21, 22–29, and ≥ 30, are taken to indicate low-mild, moderate, and severe levels of distress, respectively [29]. The K-10 has very good psychometric properties and has been widely used in a broad range of study populations, including the authors’ previous work with refugees resettled in Australia [10, 12]. Cronbach’ s alpha in the current study for this measure was high across all time points. (Time 1 α = 0.960; Time 2 α = 0.938; Time 3 α = 0.953).

### Evaluation of the program

Upon completion of the intervention (after session 4), participants also completed a series of questions assessing their experience of the program and perceived usefulness of what was taught in the program. Questions included: ‘How new was this information in the program to you’ and ‘How useful do you think the program’s information will be for you in the future?’ along with perceived usefulness of the power point presentations and activities.

### Statistical analysis

A mixed-effects model was used and the data were analysed using a combination of SPSS [30] and R (version 3.6.0) [31]. Logistic regression was used for binary outcomes. These were presented as percentages with effect sizes presented as odds ratios. Linear regression was used for continuous outcomes. These were presented as means, with effect sizes presented as mean differences. *P* values of < 0.05 were considered significant. The R functions *lme in library nlme* and *glmmPQL in library MASS* were used for the linear and logistic regression respectively. Possible covariates such as length of time in Australia, gender and marital status were examined, with only length of time in Australia found to have the strongest effect on the outcomes, and was included in all models reported in this study. All others had non-significant effects on the outcome variables, and so to avoid overfitting, were not included in the models. Multiple imputation was used to account for missing data, using predictive mean matching for continuous variables, and logistic regression for categorical variables. The R library *mice* was used to perform the multiple imputation; the number of imputations performed per analysis was 20 and the results were pooled using Rubin’s method.

## Results

### Participant flow

Figure 1 shows participant flow throughout the research stages from October 2018 to March 2019. The demographic characteristics of participants are shown in Table 2.

**Table 2:** Demographics of participants

Characteristics	N (Total = 33)	%
Gender		
Male	14	42.4
Female	19	57.6
Age in years, mean (SD)	47.7 (9.4)	–
Years of education (*n* = 24), mean (SD)	8.8 (4.1)	–
Years in Australia, mean (SD)	4.7 (3.7)	–
Years externally displaced (*n* = 32), mean (SD)	3.9 (5.7)	–
Marital Status		
Never Married	3	9.1
Married/Partner	27	81.8
Divorced	2	6.1
Widowed	1	3.0
Safety concern for family (*n* = 32)		
Not at all worried	2	6.3
A little worried	5	15.6
Quite worried	9	28.1
Extremely worried	16	50.0
K10 Psychological Distress (*n* = 32)		
Low to mild (10–21)	8	25.0
Moderate (22–30)	2	6.3
Severe (≥30)	22	68.8

Scores on measures of MHL (problem recognition, treatment and management, negative attitudes towards mental illness, help-seeking intentions) and general psychological distress (K-10) at pre-intervention, post-intervention and follow up are shown in Table 3. Table 4 presents the variables where a statistically significant relationship with increased length of time in Australia was found.

**Table 3:** Participants data across time

Variables	Pre-Intervention	Post-Intervention	3 Month Follow-up	Mean difference for post versus pre	OR for post versus pre	Mean Difference for follow-up versus pre	OR for follow-up versus pre
**Recognition of A Mental Illness**							
Problem recognised as ‘PTSD/Fear/Stress related disorder’ (95% C.I.)	54.5% (36.5–70.6)	64.5% (46.3–79.3)	56.7% (38.6–73.1)		1.65		1.06
**Treatment**							
*Actions/Activities*							
Actions/Activities concordant with PTSD recommendations thought to be helpful (mean, 95% C.I.) *Scores 9–27*	22.40 (21.3–23.5)	22.86 (21.8–23.9)	23.62 (22.5–24.7)	0.63		1.07	
Actions/Activities considered cultural and spiritual practices thought to be helpful (mean, 95% C.I.) *Scores 3–9*	7.41 (7.0–7.8)	7.14 (6.7–7.5)	7.66 (7.2–8.1)	0.23		0.24	
Percentage of participants that endorsed an action/activity thought to be most helpful which is concordant with PTSD recommendations (95% C.I.)	57.6% (40.3–73.2)	71.4% (52.1–85.2)	63.3% (44.9–78.6)		2.10		1.25
*Medications*							
Treatment with antidepressant thought to be helpful (95% C.I.)	40.6% (25.1–58.3)	43.3% (26.9–61.4)	40.0% (24.1–58.3)		0.89		1.01
Treatment with vitamins thought to be helpful (95% C.I.)	75.0% (57.1–87.1)	61.3% (43.2–76.7)	66.7% (48.1–81.2)		0.41		0.51
Treatment with Relaxants thought to be helpful (95% C.I.)	48.5% (32.0–65.3)	43.3% (26.9–61.4)	53.3% (35.6–70.3)		0.73		1.34
Percentage of participants that endorsed antidepressant considered to be the most helpful medication for the PTSD vignette (95% C.I.)	28.1% (15.2–46.1)	33.3% (18.2–53.0)	21.4% (9.8–40.5)		1.47		0.75
*Treatment Providers*							
Treatment providers concordant with PTSD recommendations thought to be helpful (mean, 95% C.I.) *Scores 3–9*	8.16 (7.8–8.5)	8.20 (7.8–8.6)	8.37 (8.0–8.7)	0.07		0.21	
Treatment providers considered to be consistent with culturally informed care thought to be helpful (mean, 95% C.I.) *Scores 2–6*	5.03 (4.7–5.3)	4.77 (4.5–5.1)	5.12 (4.8–5.4)	0.23		0.10	
**Negative attitudes towards mental illness**							
*Personal Stigma*							
‘Weak-not-sick’ (mean, 95% C.I.)	12.29 (11.4–13.1)	11.07 (10.2–12.0)	11.88 (10.9–12.8)	1.21*		0.53	
‘I would not tell’ (mean, 95% C.I.)	3.69 (3.3–4.1)	3.45 (3.0–3.9)	3.38 (3.0–3.8)	0.23		0.32	
‘Dangerous/unpredictable’ (mean, 95% C.I.)	12.22 (11.2–13.2)	13.53 (12.4–14.6)	13.63 (12.5–14.7)	1.20		1.46*	
*Social distance scale* (mean, 95% C.I.) *Scores 4–20*	11.72 (10.5–12.9)	10.07 (8.8–11.3)	9.79 (8.5–11.1)	1.62*		1.96*	
**Help-seeking Intentions**							
Percentage of participants who would approach treatment providers for help that are concordant with PTSD recommendations (95% C.I.)	54.8% (37.2–71.3)	62.1% (43.3–77.8)	56.7% (38.6–73.1)		1.37		1.05
**Psychological Distress**							
K10 score of severe distress (95% C.I.)	68.7% (50.8–82.4)	63.3% (44.9–78.6)	37.9% (22.2–56.7)		1.49**		7.47**
K10 total score (mean, 95% C.I.)	33.63 (29.8–37.5)	31.73 (27.7–35.7)	29.21 (25.1–33.3)	1.90		3.00*	

*P < 0.05, **P ≤ 0.001

**Table 4:** Impact of Length of time in Australia

Variable	Mean increase for a one-year increase of time spent in Australia	Odds ratio for a one year increase of time spent in Australia
Treatment with antidepressant thought to be helpful	0.44*	
Antidepressant considered to be the most helpful medication	0.36*	
K10 score of severe distress		66.55**
K10 total score	1.79*	

*P < 0.05, **P ≤ 0.00

### Mental health literacy

#### Problem recognition

Approximately half (54.5%) of participants recognised PTSD/fear/stress-related disorder as the main problem described in the vignette pre-intervention. This improved to 64.5% immediately post-intervention but decreased to 56.7% at follow up (3 months post-intervention). These changes were not statistically significant at either time point.

#### Treatment actions/activities

Participants appeared likely to endorse actions/activities deemed to be concordant with evidence-based treatment of PTSD as helpful immediately following the intervention (22.40 versus 22.86) and a further increase (23.62) was observed at follow up. This was also accompanied by an increase in the proportion of participants endorsing a concordant action/activity as the most helpful between pre- (57.6%) and post- (71.4%) intervention. The perceived helpfulness of practices deemed to be culturally and spiritually informed appeared to decrease from pre- intervention to post-intervention (7.41 versus 7.14) but increased to above pre-intervention scores (7.66) at follow up. None of these changes was statistically significant.

#### Medications

From pre- to post-intervention, there was a slight increase in the endorsement of antidepressants as helpful in the treatment of PTSD (40.6% initially versus 43.3%) and of recognition that relaxants were harmful between pre- (48.5%) and post- (43.3%) intervention. However, these changes were not statistically significant, and were not maintained at follow-up. Finally, it was noted with increasing length of time living in Australia, there was a statistically significant increase in the perceived helpfulness of antidepressant medication in the treatment of PTSD.

#### Treatment providers

There was an increase in the perceived helpfulness of treatment providers deemed to be concordant with evidence-based treatment of PTSD from pre-intervention to post-intervention (8.16 versus 8.20) and from post-intervention to follow-up (8.20 versus 8.37), while the perceived helpfulness of treatment providers deemed to be providing culturally and spiritually informed care decreased from pre-intervention to post-intervention (5.03 versus 4.77) but increased between post-intervention and follow up (4.77 versus 5.12). None of these changes were statistically significant.

#### Negative attitudes towards mental illness

There was a statistically significant decrease in the belief that the character in the vignette was ‘weak-not-sick’ (personal stigma) from pre- to post-intervention (12.29 versus 11.07; *p* < .05). A further decrease, which was not statistically significant, was observed from post-intervention to follow-up 12.29 versus 11.88). Additional decreases were observed in the ‘I would not tell anyone’ subscale from pre- to post-intervention (3.69 versus 3.45) and from post-intervention to follow-up (3.45 versus 3.38). However, there was an increase in the ‘dangerous/unpredictable’ subscale from pre- to post-intervention (12.22 versus 13.53). None of these changes were statistically significant; however, an increase in scores on the ‘dangerous/unpredictable’ subscale between pre-intervention and follow-up (12.22 versus 13.63; *p* < .05) was statistically significant.

Finally, statistically significant decreases on the social distance scale scores indicating a greater willingness to spend time with Dawood/Mariam were observed both from pre-intervention to post-intervention (11.72 versus 10.07; *p* < .05) and from pre-intervention to follow-up (10.07 versus 9.79; *p* < .05).

### Help-seeking intentions

The proportion of participants who indicated that they would approach treatment providers deemed to be providing evidence-based treatment, were they to have a problem such as the one described in the vignette, increased from 54.8 to 62.1% from pre- to post-intervention (56.7% at follow up). These changes were not statistically significant.

### General psychological distress

There was a statistically significant decrease in the proportion of participants reporting severe levels of distress (K-10 ≥ 30) from pre-intervention (68.7%) to post-intervention (63.3%; *p* < .05) and from pre-intervention to follow up (37.9%; *p* < .01). From Table 4, it can be noted that greater time in Australia was significantly associated with higher levels of distress, as measured by both the K-10 total score (*p* < .05) and the proportion of participants with severe distress (*p* < .01).

### Evaluation of the program

**Fig. 2:**
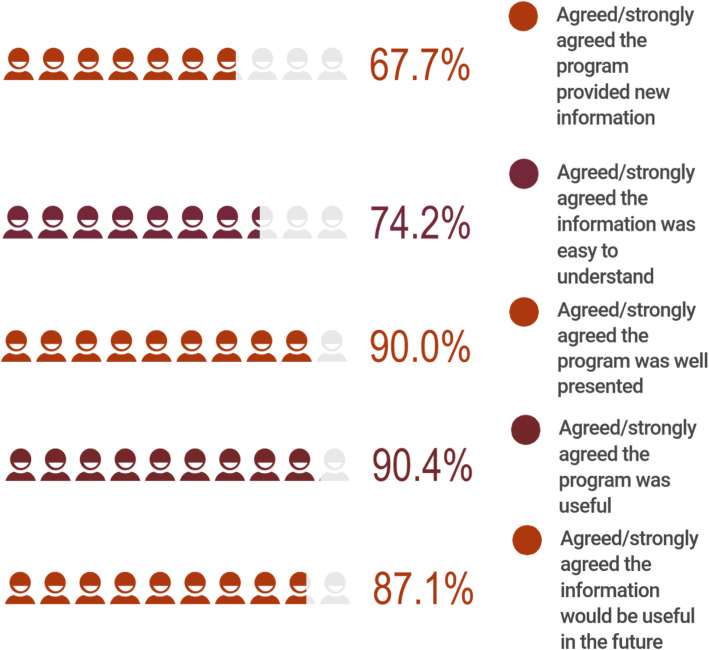
Participants evaluation of the Mental Health Literacy Program

Figure 2 shows participant evaluation of the program, which was completed as part of the post intervention survey. The evaluation indicated that participants’ experience of the Mental Health Literacy Program overall was positive, with participants expressing that the information provided was new, easy to understand, useful and well-presented.

## Discussion

We conducted a preliminary trial of a mental health promotion program designed to improve aspects of MHL and related variables among Arabic-speaking refugees resettled in an urban area of Sydney, Australia. An uncontrolled, pre-post design was employed in which assessments were conducted immediately prior, immediately following, and 3 months following program implementation. Immediately following the intervention, there were non-significant improvements in most aspects of MHL assessed, including problem recognition, positive beliefs about the use of interventions most likely to be helpful, stigmatising attitudes and beliefs, and help-seeking intentions. In most cases these improvements were sustained at follow-up. However, only changes relating to stigmatising attitudes, and a reduction in perceived need for social distance in particular, reached statistical significance. There was also a statistically significant decrease in participants’ levels of general psychological distress following the intervention, and this change was sustained at follow-up.

The study findings need to be interpreted with caution, given that this was a preliminary trial with a relatively small sample size and with no control group. Nevertheless, it is encouraging that positive changes in most aspects of MHL assessed were observed, that these changes tended to be sustained at follow-up and that effect sizes for at least some of the changes were large enough for statistical significance to be reached despite sample size. When comparing these findings to similar previous interventions in resettled refugee populations, the observed reductions in stigmatising attitudes in particular are encouraging, given that both Sanhori et al. [15] and Subedi et al. [17] were unable to demonstrate reductions in these attitudes, despite having larger sample sizes. Indeed, reductions in stigma have proven challenging even in the general population [32]. The relatively intensive nature of the intervention employed in the current study, the use of bilingual project staff, delivery of the intervention face-to-face rather than via telephone or online [33], and the fact that most participants in the current study (68.8%) had high baseline levels of general psychological distress, may all have contributed to the relatively positive results in this regard [14, 32, 33]. It is possible that even stronger results would have been observed had it been possible to build consumer involvement and contact (that is, someone with a diagnosed mental illness such as PTSD) into the intervention. To our knowledge, the potential benefits of consumer contact in improving the MHL of refugee populations has not yet been examined, despite evidence from general population studies suggesting that contact of this kind is beneficial in reducing stigmatising attitudes towards individuals suffering from at least certain mental health problems [34]. Hence, this would be of interest in future research.

While increased awareness of the potential benefits of medication in the treatment of PTSD following the intervention was among the changes that did not reach statistical significance in the study, a statistically significant positive association was observed between the perceived helpfulness of antidepressant medication in the treatment of PTSD and time spent living in Australia. This finding is notable given that poor awareness and understanding of the use of psychotropic medication in the treatment of PTSD and comorbid mental health problems is a known barrier to compliance among resettled refugees taking this medication [35]. Greater exposure to and education about the Australian health system with time may be a factor in this association. Participants in the current study had lived in Australia for 5 years on average. Hence, relatively greater familiarity with the Australian health system among participants in the study, including the role played by general practitioners in the treatment of mental health problems, may also account for the relatively high proportion of participants at baseline who endorsed the use of interventions for PTSD consistent with current guidelines [24].

Arguably the most notable finding of this study is that the intervention was associated with marked improvement in participants’ actual mental health, as measured by the Kessler Psychological Distress scale, from baseline to follow-up. Previous research has demonstrated that higher distress levels were associated with longer resettlement periods amongst Iraqi refugees in Australia [7] and this was also noted in this study. Some postulated contributors to such elevated distress levels have been noted to include racism, perceived discrimination and cultural bereavement [36] and are beyond the scope of the current intervention. However, the ability to provide participants with skills in reducing levels of arousal and reactivity to negative emotions through the mindfulness practices is important and should not be discounted. Nonetheless, given participants’ high levels of distress at baseline, the potential for regression to the mean and the lack of a control group, attribution of this change to the intervention is problematic. It is also possible that the improvements in psychological distress may have been attributable to the act of coming together on a regular basis; however given that most participants had been attending the English college for some time prior to the intervention, this seems less likely. Finally, it is also not possible to know which changes in MHL, if any, were more or less conducive to the observed reductions in levels of distress. It is worth noting, however, that the self-help strategies delivered in the final session of our program were derived from a recently evaluated mindfulness intervention, also delivered entirely in Arabic over a 5-week period, and which also found marked improvements in general psychological distress among Arabic-speaking refugees both immediately following and 12 weeks following the intervention [37]. Participants in that study, like those in the current study, agreed that mindfulness was congruent with their religion, culture and way of life and this was likely a factor in the positive feedback participants provided about the program [16, 37]. Our findings are also consistent with findings noted by Pandya [17] where the mental wellbeing of those participants who self-practiced mindfulness exercises demonstrated most improvement. A pre-post comparison of the reported use of these strategies and their perceived helpfulness over time would be a useful addition in future research of this kind. The finding that levels of distress were positively correlated with time spent in Australia among participants in the current study, which is consistent with previous research in Iraqi refugees [7], may reflect an increasingly adverse impact of post-migration stressors on mental health over time [38]. It highlights the importance of mental health programs for refugees being implemented as soon after resettlement as possible. As we have argued elsewhere [12], mental health promotion programs, which target the refugee community as a whole, would ideally be integrated with early intervention programs that target individuals with symptoms of PTSD and related conditions.

As this was a pilot study, sample size was small, thereby limiting statistical power to detect pre-post change, and a quasi-experimental (uncontrolled, pre-post) study design was employed. Funding limitations precluded the ability to recruit a control group. Clearly a larger, controlled trial, in which the effect of modifiers and mediators can also be considered, will be an important next step. While attrition was low in the current study, missing data were problematic in some sections of the survey. In future, online or interviewer-administered surveys could reduce missing data. Recruitment of participants via the Adult Migrant English Program (AMEP) may have detracted from sample representativeness, however it should be noted that the AMEP is a core component of the resettlement program for humanitarian migrants to Australia. Finally, the possibility of selection bias with those committed to improving their mental wellbeing agreeing to participate in the study cannot be discounted and in future, investigations employing a randomised approach should be undertaken. Strengths of the current research include the availability of all program and study materials in Arabic and the use of bilingual staff to facilitate program presentation and data collection [14]. No doubt attention to these details was a factor in the program being well-received and in attrition being lower than in other studies [16]. Longer term, the program could be expanded to other refugee populations and more broadly disseminated, for example via other AMEP centres.

## Conclusion

While further research, employing a more rigorous study design and larger sample size, will be needed, results of this initial trial suggest that a culturally tailored mental health promotion program targeting key aspects of mental health literacy can improve the mental health of Arabic-speaking refugees resettled in a Western nation.

## Data Availability

We would like to acknowledge that data from each participant from this study cannot be shared in order to comply with the Western Sydney University Ethics policy.

## Abbreviations

UNHCR: United Nations High Commission for Refugees
ABS: Australian Bureau of Statistics
PTSD: Post-Traumatic Stress Disorder
MHL: Mental Health Literacy
MHFA: Mental Health First Aid
AMEP: Adult Migrant English Program
K10: Kessler Psychological Distress Scale
